# Sex impacts outcome in minimally invasive surgery of the ascending aorta: a propensity score matched analysis

**DOI:** 10.1186/s13019-026-04274-8

**Published:** 2026-06-02

**Authors:** Florian Helms, Heike Krüger, Ezin Deniz, Andreas Martens, Aron-Frederik Popov, Bastian Schmack, Jan Dieter Schmitto, Alexander Weymann, Arjang Ruhparwar, Morsi Arar

**Affiliations:** 1 Division for Cardiothoracic-, Transplantation- and Vascular Surgery, Hannover Medical School, Carl-Neuberg-Str. 1, Hannover, 30625 Germany; 2 Clinic for Cardiac Surgery, University Clinic Oldenburg, Oldenburg, Germany; 3 Clinic for Cardiac Surgery, Asklepios Clinic Harburg, Hamburg, Germany

**Keywords:** Aortic surgery, Ascending aorta, Aortic valve, Minimally invasive techniques, Sex-specific analysis

## Abstract

**Background:**

Minimally invasive approaches have gained immense importance in surgery of the aortic valve, aortic root, and ascending aorta over the last decades. Despite this, data concerning impact factors of the postoperative outcome and especially investigations regarding sex-specific outcome parameters for minimally invasive aortic surgery are still lacking to date.

**Methods:**

We present a single-center analysis of 387 patients undergoing supracoronary ascending aorta replacement, Wheat procedure, David procedure, or Bentall procedure through a minimally invasive access. A multivariate linear model was developed to identify predicting factors for a prolonged intensive care unit stay. Subsequently, the impact of the patients sex on perioperative complications and outcome as well as short- and long-term survival was investigated using a propensity score matched analysis of each 118 women and men undergoing minimally invasive ascending aortic procedures.

**Results:**

Female sex, patients age at operation, and operation times were identified as independent patient-specific predictors for ICU length of stay after minimally invasive ascending aortic surgery. The perioperative stroke-rate was significantly higher in women compared to men (7.6% vs. 1.7%, *p* = 0.031). Erythrocyte concentrate transfusion requirement was significantly higher in females (4 (IQR 2–5) vs. 2 (QR 0–4), *p* < 0.001). No significant differences were found between male and female patients with respect to short- and long-term survival.

**Conclusions:**

Sex impacts outcome after minimally invasive ascending aortic surgery. In particular, female patients had worse short-term outcome compared to men with respect to perioperative stroke, ICU length-of-stay, and transfusion requirements.

## Background

Minimally invasive techniques have gained immense importance in cardiovascular surgery over the last decades. Therefore, numerous advances in access strategies [1], cannulation techniques [2, 3], and perioperative care [4] have been made in recent years. These developments allowed the implementation of minimally invasive cardiac surgery for numerous valve- and coronary operations. Particularly for operations of the aortic valve, the minimally invasive approach via upper partial sternotomy has been proven to shorten the intensive care- and hospital stay times and reduce blood transfusion rates [5, 6]. Following this evidence, the minimally invasive approach has been extended towards operations of the ascending aorta too [1]. After satisfactory results of minimally invasive isolated ascending aorta replacement, the indication has been extended towards more complex procedures involving the aortic valve and aortic root over the last decades [7–9]. In this context, the minimally invasive David procedure [10], Wheat procedure [11], and Bentall procedure [12] have been successfully established and described. While satisfactory short-term results have been reported for each of these entities, previous studies did not take subgroup-specific characteristics, e.g. sex or age, of the patient clientele undergoing minimally invasive ascending aortic surgery into account. Particularly, no specific analysis of the influence of male versus female sex on the short- and long-term outcome after minimally invasive ascending aortic surgery has been performed to date. However, studies investigating the effect of gender on outcomes of full-sternotomy cardiac surgical procedures demonstrated significant differences between male and female sex concerning the surgical success rate and short-term outcome, often with more unfavorable outcome in women compared to men [13]. Consequently, patient sex may play a decisive role in minimally invasive procedures of the ascending aorta as well. While the current EACTS/STS guidelines for diagnosing and treating acute and chronic syndromes of the aortic organ include a specific chapter for aortic diseases in women, no sex-specific intervention threshold or treatment recommendations were given for the ascending aorta – arguably due to the lack of sex-specific data on the surgical risk. All of the above mentioned factors call for a specific investigation of the impact of sex on the outcomes after minimally invasive aortic surgery.

In this study, we provide a single-center analysis of patients undergoing minimally invasive surgery of the ascending aorta identifying independent risk factors for prolonged intensive care unit (ICU) stay time. Further, we report a propensity score matched analysis of the outcomes of women versus men undergoing minimally invasive procedures of the ascending aorta.

## Methods

### Patients and data collection

A total of 387 consecutive patients with aneurysms of the ascending aorta undergoing minimally invasive supracoronary ascending aorta replacement (*n* = 86, 22.2%), Wheat procedure (*n* = 95, 24.5%), David procedure (*n* = 94, 24.3%), or Bentall-procedure (*n* = 112, 28.9%) from 2000 to 2024 were included in a retrospective single-center analysis. As part of the standard preoperative diagnostic protocol, duplex carotid artery sonography was performed in every patient prior to scheduling the surgery, in order to rule out any carotid pathology requiring intervention. The patients evaluated for inclusion in this study were therefore free of interventionally relevant carotid stenoses. Data concerning preoperative and intraoperative parameters as well as short-term perioperative complications were collected prospectively in our clinical database. Perioperative stroke was defined as a radiographically confirmed ischemic cerebral lesion occurring during operation or within 30 days after the operation. Long-term survival was investigated with continuous prospective follow-up by our outpatient clinic. In the context of this study, the patients sex was defined as the biological sex of the patients. Data collection and analysis was conducted in accordance with the ethics board approval by the ethics commission of Hannover Medical School, Approval No. 11461_BO_K., June 20th 2024.

### Surgical technique

All procedures included in this study were performed via an upper J- shaped partial sternotomy. As no involvement of the aortic arch was present in the patients included in this study, direct cannulation of the aorta as far distally as possible toward the aortic arch and direct right atrial cannulation were performed via the sternotomy incision. The left ventricular vent was placed via the right upper pulmonary vein. Carbon dioxide insufflation was initiated prior to transection of the aorta and was continued throughout the aortic cross-clamp period. Depending on the aneurysm localization and extent as well as the aortic valve function, valve morphology, and involvement or non-involvement of the aortic root, the according procedure type was chosen. For isolated supracoronary aortic aneurysms without aortic valve pathologies, supracoronary aortic replacement was performed using a straight tube graft. In patients with structural aortic valve pathologies and supracoronary aortic aneurysms without aortic root involvement, combined aortic valve implantation und supracoronary ascending aorta replacement (i.e. Wheat procedure) was chosen. In root aneurysms without structural aortic valve pathologies, aortic root reconstruction (i.e. David procedure) was performed, and for combined structural aortic valve and root pathologies, valve-bearing composite conduit implantation (i.e. Bentall procedure) was used. The valve type (mechanical versus biological prosthesis) was chosen on a individual patient specific basis taking into account patient age, comorbidities and patient’s preferences. Following the respective procedure, careful deairing was carried out. Intraoperative transoesophageal echocardiology was used during deairing to visualize residual intracardiac air and confirm complete deairing prior to weaning the patient from cardiopulmonary bypass, and the wound was closed in standard fashion. The patients were then transferred to our cardiac intensive care unit for further stabilization.

### Data analysis

**Fig. 1:**
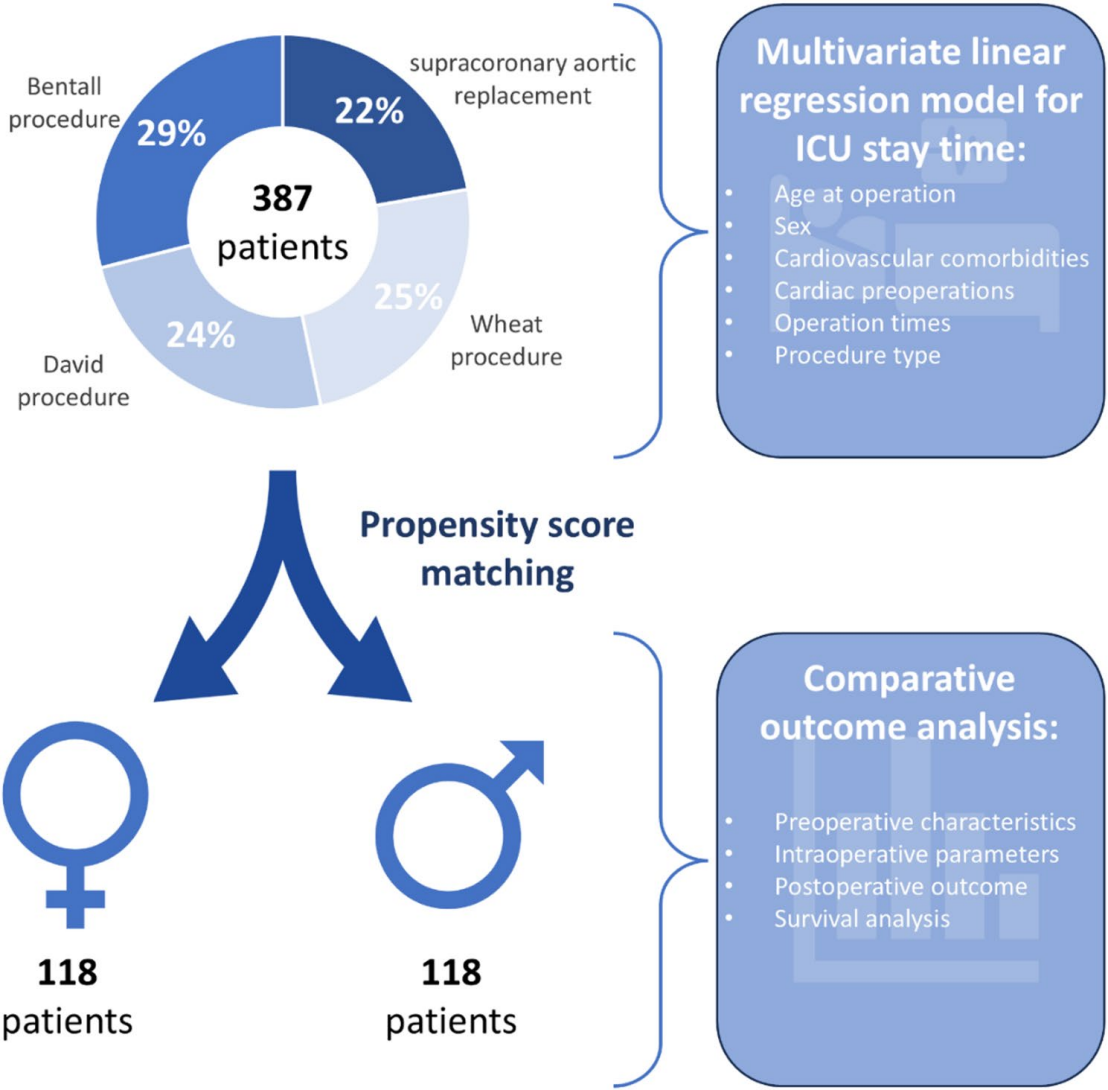
Study Flow-chart. Schematic overview of the study protocol. In a first step, the overall patient cohort was analyzed for risk factors for longer ICU-stay times using a multivariate linear model. In the second step, propensity score matching identified 118 pairs of women and men, which were compared for pre-, intra-, and postoperative variables

Data analysis was performed in two steps (Fig. 1): First, a multiple linear regression model was created to identify independent risk factors for intensive care unit stay time. Subsequently, a propensity score matched analysis of the intraoperative and perioperative short-term outcome and long-term survival of female versus male patients was performed.

In the multiple linear regression model, the available factors concerning the preoperative patients characteristics as well as intraoperative variables and operation times were evaluated. Specifically, the factors age at operation, sex, procedure types (ascending aorta replacement, Wheat procedure, David procedure, and Bentall procedure), and operation-, bypass-, and cross-clamp- times as well as preoperative comorbidities including chronic obstructive pulmonary disease (COPD), coronary artery disease, history of stroke events, bicuspid aortic valve, Marfan syndrome, and cardiac preoperations were included as potential predictors for the ICU stay time. Model fitness and explained variance were analyzed using the multiple correlation coefficient R and the multiple determination coefficient (R²). Pearson’s correlation and tolerance/variance influence factor analysis were used to test for possible multicollinearity of the predictors.

After female sex was identified as an independent risk factor for ICU stay time, further investigation of potential differences in intra- and perioperative characteristics and complications between male and female patients were conducted. For this, propensity score matching was performed. Here, the above-mentioned patient characteristics and preoperative comorbidities (i.e. patients age at operation, COPD, coronary artery disease, preoperative stroke events, bicuspid aortic valve, Marfan syndrome, and cardiac preoperations) as well as the procedure type were included as variables in the propensity score matching protocol. Matching was performed as one-on-one nearest neighbour propensity score matching with a match-tolerance factor of 0.2.

### Statistics

Statistical analyses were performed using IBM SPSS Statistics 29 (IBM Corp., Armonk, NY, USA, 1989, 2021). The Shapiro-Wilk test was used to test continuous variables for normal distribution. Normally distributed data are given as mean ± standard deviation and were compared using the T-test, data not meeting normal distribution criteria are given as median and interquartile range and were compared using the Mann-Whitney test. Categorial variables are reported as absolute numbers (n) and percentages. Long term survival was analyzed using the Kaplan-Meier analysis and survival comparison was done by Log-rank testing. Differences were considered significant at *p* < 0.05.

## Results

### Predictors for ICU length of stay

For identification of patient characteristics and operative factors predicting ICU length of stay, a multivariate linear model was created. With Cohen’s R of 0.394 and Cohen’s R² of 0.155, moderate goodness of fit was identified for the overall model. ANOVA revealed that the factors included in the overall model significantly predicted ICU stay time: F(7, 380) = 9.798, *p* < 0.001). For the tested variables, variance inflation factors were < 5 indicating overall low to moderate risk of multicollinearity with the exception of operation- time and cross-clamp times, which naturally correlate with each other. Within the model, patient sex, age at operation, and operation time were statistically significant positive predictors of the ICU stay time, while conduction of the Wheat procedure was identified as a negative predictor of ICU length of stay (Table 1).

**Table 1:** Multivariate linear regression model

Predictor	Regression- coefficient B	95% CI	*p*- value	Tolerance	VIF
Sex (female)	0.668	0.11–1.32	**0.046**	0.792	1.263
Age (years)	0.032	0.01–0.06	**0.010**	0.598	1.672
COPD	0.174	−0.90–1.25	0.751	0.962	1.040
CAD	0.263	−0.50–1.03	0.491	0.927	1.079
Preoperative stroke	0.301	−1.58–2.19	0.753	0.927	1.079
Bicuspid aortic valve	0.346	−0.31–1.00	0.300	0.710	1.408
Marfan syndrome	0.447	−1.21–2.11	0.596	0.840	1.190
Cardiac preoperation	2.020	−1.26–5.30	0.227	0.904	1.106
Operation time (min)	0.013	0.002–0.024	**0.018**	0.152	6.658
Bypass time (min)	0.006	−0.001–0.014	0.092	0.253	3.905
Cross-clamp time (min)	0.007	−0.021–0.007	0.336	0.195	5.117
Procedure (Wheat)	−0.927	−1.80 – −0,05	**0.038**	0.598	1.671

CI = confidence interval, VIF = variable inflation factor, COPD = Chronic obstructive pulmonary disease, CAD = Coronary artery disease

Statistically significant values are marked in bold

### Impact of sex on short-term outcome: propensity score matched analysis

Following these findings, the impact of sex on intra- and postoperative outcome parameters was further investigated by direct comparison of female versus male patients undergoing minimally invasive procedures of the ascending aorta. In the overall study population, 67.4% (*n* = 261) were male and 32.6% (*n* = 126) were female. When comparing the age at operation of the two groups in the unadjusted population, women included in the study were significantly older compared to men (65.4 ± 12.7 years versus 55.5 ± 14.1 years, *p* < 0.001). Propensity score matching identified 118 matches (236 patients in total), representing 61% of the overall study population, whereby 93.6% of women in the initial study population were included.

Mean age after propensity score matching was similar in both groups with 63.3 ± 11.5 in women versus 65.1 ± 12.9 years for men (*p* = 0.253). After matching, preoperative patient characteristics did not differ significantly (Table 2).

**Table 2:** Preoperative characteristics

Characteristics	Female	Male	*p*- value	SMD
Age [years]	63.3 ± 11.45	65.1 ± 12.9	0.253	−0.148
BMI (kg/m²)	23.0 ± 4.9	24.6 ± 4.8	0.130	−0.330
COPD	8.5% (*n* = 10)	8.5% (*n* = 10)	1.000	0
CAD	18.6% (*n* = 22)	18.6% (*n* = 22)	1.000	0
Preoperative stroke	4.2% (*n* = 5)	1.7% (*n* = 2)	0.446	0,113
Bicuspid aortic valve	27.1% (*n* = 32)	39.0% (*n* = 47)	0.053	−0,212
Marfan syndrome	2.5% (*n* = 3)	1.7% (*n* = 2)	0.317	0,044
Cardiac preoperation	0% (*n* = 0)	0.8% (*n* = 1)	0.316	−0,127

SMD = Standardized mean differences, BMI = Body mass index, COPD = Chronic obstructive pulmonary disease, CAD = Coronary artery disease

In the intraoperative characteristics, longer operation-, bypass-, and cross-clamp times were observed in men compared to women. Contrary to that, the need for intraoperative erythrocyte concentrate transfusion was significantly higher in women than in men (Table 3). Conversion rates and intraoperative thrombocyte as well as fresh frozen plasma transfusion did not differ significantly between the groups. Total ventilation time was longer in women with 11.9 h (IQR 8.9–16.5 h) compared to men with 10.8 h (IQR 7.5–14.0 h, *p* = 0,048).

**Table 3:** Intraoperative characteristics

Characteristics	Female	Male	*p*- value
Conversion	1.7% (*n* = 2)	2,5% (*n* = 3)	0.651
Concomitant ACB	0.8% (*n* = 1)	1.7% (*n* = 2)	0.561
Operation time [min]	225 (190–269)	243 (202–299)	**0.038**
Bypass time [min]	127 (104–161)	138 (109–176)	0.110
Cross-clamp time [min]	72 (51–101)	85 (63–110)	**0.019**
EC intraop. [n]	2 (1–4)	0 (0–2)	**< 0.001**
TC intraop. [n]	2 (0–2)	2 (0–2)	0.394
FFP intraop.[n]	0 (0–3)	0 (0–3)	0.223

ACB = Aorto-coronary bypass grafting, EC = Erythrocyte concentrate, TC = Thrombocyte concentrate, FFP = Fresh frozen plasma

Statistically significant values are marked in bold

Considering postoperative complications, a distinctive difference in the perioperative stroke rate was observed: With a stroke rate of 7.6% (*n* = 9), women suffered postoperative stroke events more frequently compared to men with 1.7% (*n* = 3), *p* = 0.031 (Table 4). Concerning hospital stay as well as further investigated postoperative complications, no statistically significant sex-specific differences were found. Nonetheless, it is noticeable that postoperative extracorporeal membrane oxygenation (ECMO)-support was needed in three cases among women (2.5%) in the matched population, while no case occurred among men, although this difference did not reach statistical significance (*p* = 0.081). Congruent with the intraoperative transfusion requirements, the overall need for transfusion was higher in women compared to men (*p* < 0.001).

**Table 4:** Postoperative characteristics

Characteristics	Female	Male	*p*- value
Ventilation time (h)	11.9 (8.9–16.5)	10.8 (7.5–14.0)	**0.048**
Hospital stay (d)	10 (8–13)	10 (8–13)	0.358
Stroke	7.6% (*n* = 9)	1.7% (*n* = 2)	**0.031**
Re-thoracotomy (early)	4.2% (*n* = 5)	2.5% (*n* = 3)	0.472
Re-thoracotomy (late)	0.8% (*n* = 1)	0.8% (*n* = 1)	1.000
Wound infection	0.8% (*n* = 1)	0% (*n* = 0)	0.316
Tracheostomy	2.5% (*n* = 3)	0.8% (*n* = 1)	0.313
Dialysis	0.8% (*n* = 1)	0.8% (*n* = 1)	1.000
Reanimation	2.5% (*n* = 3)	1.7% (*n* = 2)	0.651
ECMO postop.	2.5% (*n* = 3)	0% (*n* = 0)	0.081
Overall EC transfusion	4 (2–5)	2 (0–4)	**< 0.001**
Overall TC transfusion	2 (0–2)	2 (0–2)	0.137
Overall FFP transfusion	0 (0–3)	0 (0–3)	0.647

ECMO = Extracorporeal membrane oxygenation, EC = Erythrocyte concentrate, TC = Thrombocyte concentrate, FFP = Fresh frozen plasma. Re-thoracotomy (early) = re-thoracotomy within 24 h postoperatively, Re-thoracotomy (late) = re-thoracotomy > 24 h postoperatively

Statistically significant values are marked in bold

### Survival analysis

**Fig. 2:**
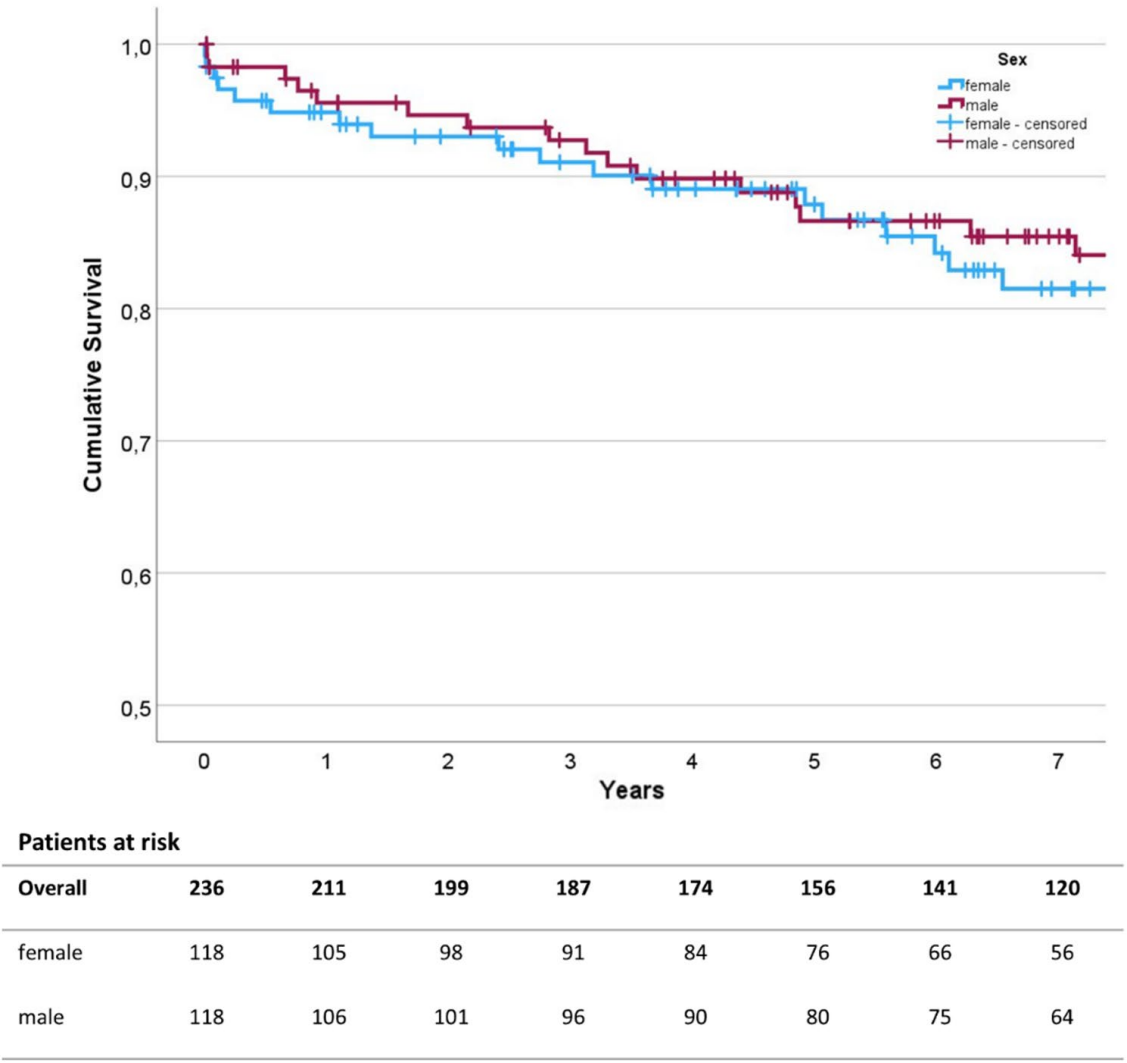
Survival analysis. Survival was analyzed using the Kaplan-Meier survival curves up to 7 years post-operation. The numbers of patients at risk are given for each year of follow up

Overall survival analysis was performed using the Kaplan-Meier survival curves (Fig. 2). Log-Rank testing revealed no statistically significant difference in long-term survival between the groups (*p* = 0.740). Moreover, 30 day mortality did not differ significantly between with 2.5% (*n* = 3) in women and 1.7% (*n* = 2) in men (*p* = 0.651).

## Discussion

The main findings of this study can be summarized as follows: (I) Female sex and age were identified as independent patient-specific predictors for ICU length of stay after minimally invasive ascending aortic surgery. (II) The perioperative stroke-rate was significantly higher in women compared to men. (III) Erythrocyte concentrate transfusion requirement was significantly higher in females. (IV) No significant differences were found between male and female patients with respect to short- and long-term survival.

Overall, sex-specific differences are known to play a role in aortic diseases and surgery: In a previous report, we found sex-specific differences in risk factors for early mortality in patients undergoing thoracoabdominal aortic replacement [14]. Further differences in the epidemiology, pathophysiology, and outcomes of aortic disease between women and men were reviewed by Huckaby et al. [15]. In the multivariate linear regression model, operation time, and the patient age at the time of operation were predictors for the length of the ICU stay. These findings were both expected and are congruent with previous reports on risk factors for a prolonged stay in intensive care, in which the factors age and operation- or bypass- time were also identified as relevant factors for the prediction of intensive care length of stay after conventional cardiac procedures [16, 17]. However, the multivariate linear model also revealed that female sex was an independent predicting factor for ICU stay time after minimally invasive procedures of the ascending aorta. No previously reported original data exists for the impact of sex on ICU length of stay after minimally invasive aortic surgery. Available reports on the impact of sex on ICU stay times are mainly retrospective database or registry analyses focusing on valvular or coronary patients operated via full sternotomy. Here, a registry analysis of the Society for Thoracic Surgery (STS) database on coronary revascularization reported by Dassanayake et al. also showed that female sex was associated with prolonged ICU length of stay [18]. In a further national database analysis of the National Adult Cardiac Surgery Audit from the United Kingdom, Dixon et al. found prolonged overall hospital stay times in women compared to men after isolated or combined valvular or coronary cardiac surgery, while the length of the ICU stay was not investigated specifically in that data analysis [19]. In contrast to that, overall hospital stay times did not differ significantly in our comparative study after propensity score matching. This dissensus may be due to an increased burden of diseases and preoperative comorbidities in women compared to men in the unmatched database analyses, which has been shown to potentially bias the registry analyses [20].

Following the finding that female sex was predictive for prolonged ICU stay, further investigations of the pre-, intra-, and postoperative factors and complications that may explain this phenomenon were conducted. Since the patient age at operation was significantly higher in women compared to men in the initial study population, propensity score matching was performed to exclude potential age bias. Correlating with the findings of the regression analysis, total ventilation times were longer in women compared to men after propensity score matching. In the direct comparison of women versus men undergoing minimally invasive ascending aortic surgery, the stroke rate was distinctively and statistically significantly higher in women with 7.6% (*n* = 9) compared to men with 1.7% (*n* = 2). As far as can be determined from the records of the anaesthesiology, perfusion, and surgical documentation, intraoperative management and surgical technique did not differ between female and male patients in the present cohort. Thus, the observed difference may be more likely due to patient-inherent factors rather than procedural differences. Again, available literature concerning postoperative outcomes of women versus men is highly limited and contradicting findings can be identified in current reports: Congruent to our findings, a systematic review and meta-analysis of 30 primary research articles comparing postoperative short-term sex-specific outcome after coronary or valvular surgery found that women were at higher risk for postoperative stroke with a cumulative Odds-ratio of 1.2 (CI 1.07–1.34) [13]. Contrary to that, a German registry analysis of patients undergoing surgical aortic valve replacement identified female sex as a protective factor against postoperative stroke with an adjusted Odds Ratio of 0.61 [21]. Dixon et al. explain the higher stroke rate observed in their meta analysis with a potentially higher preoperative burden of arteriosclerotic diseases in women compared to men, which may serve as a risk factor for thromboembolic stroke events [13]. However, in our study no significant differences were found between men and women concerning preoperative coronary disease, which may function as a limited surrogate parameter for general arteriosclerotic disease. With respect to perioperative hemodynamic instability, which, mediated by increased vasopressor use and cerebral vasoconstriction, may be associated with a higher perioperative stroke rate, three female patients required postoperative circulatory support, while no male patient required ECMO- therapy. This surrogate parameter may indicate inequal conditions between female and male patients concerning perioperative hemodynamic stability which may in part be associated with the observed discrepancies in the stroke rates. After adjusting for inequal baseline characteristics, Kaier et al. [21] found lower stroke rates in women compared to men, while there were no significant differences in stroke rates prior to adjustment for preoperative baseline patient characteristics. This again suggests that inequal burden of disease between women and men may be a plausible explanation for increased stroke risk as it was observed in our study as well. However, due to the retrospective character and study design, no causal relations can be identified from this data analysis.

While the transfusion requirement is usually reported to be lower in minimally invasive cardiac surgery compared to classical full-sternotomy cases [5, 6], distinct sex-specific differences in red blood cell transfusion requirements were observed between women and men undergoing minimally invasive ascending aortic surgery: With a median overall transfusion requirement of four (IQR 2–5) versus two (IQR 0–4) concentrates, female patients received significantly more erythrocyte concentrate perfusions compared to male patients. This difference was also present concerning the intraoperative blood cell transfusion rates even despite significantly lower operation-, bypass-, and cross-clamp- times observed in the female versus the male study population. This observation is consistent with a single-center analysis report by Ränsänen et al., that investigated risk factors for red blood cell transfusion after conventional cardiac surgery [22]. They even identified female sex as an independent risk factor for blood cell transfusion after conventional cardiac surgery. Furthermore, Wang et al. observed higher erythrocyte concentration transfusion rates in women compared to men after coronary artery bypass grafting as well [23]. According to our findings, this correlation of female sex and higher red blood cell transfusion requirements is also consistent in minimally invasive ascending aortic surgery. Although preoperative hemoglobin levels and anemia rates were not systematically assessed in the context of this study, differences in this aspect may also play a role in the transfusion requirements after minimally invasive ascending aortic surgery: Based on external evidence, lower overall blood volume in women and a higher rate of preoperative anemia may influence blood cell transfusion requirements in women compared to men during and after cardiac surgery [24].

In the survival analysis, no significant differences were found between male and female patients for both, 30-day mortality and in the long-term survival up to seven years postoperatively (Fig. 2) despite the differences in postoperative morbidity and length-of-stay. This is in contrast to previous reports concerning the impact of perioperative stroke on postoperative survival outcomes. As an example, Karunanantham et al. reported highly elevated short- and long-term mortality in patients with perioperative stroke in cardiac surgery [25]. This discrepancy may possibly be explained by the fact that, even though the rate was significantly higher among women, patients who suffered perioperative stroke were only a minor part of the overall patient population, which may explain why the potential effects of the higher stroke rates did not have a detectable impact on overall survival or length of hospital stay. In contrast to the similar overall long-term survival found in this study, the currently available literature on sex-specific analyses of cardiac surgery paints a very different picture: Here, numerous reports state worse short- and long term survival rates in women compared to men [16, 17, 26]. As discussed in each of the above-mentioned studies, one of the main factors for lower survival rates in women compared to men observed in the registry studies may be differences in preoperative characteristics such as the higher overall burden of disease, higher age, or later presentation due to atypical symptom presentation in women. After correcting for preoperative characteristics, Dalén et al. found no significant differences between men an women after coronary bypass grafting, which again is conclusive with our study results on minimally invasive ascending aortic surgery [20].

### Limitations

Due to the study design as a retrospective single-center analysis, the overall case number was limited. The cumulative analysis of different minimally invasive procedures of the ascending aorta (i.e. isolated ascending aorta replacement, Wheat-, Bentall-, and David- procedure) carried the risk of higher variance and potential bias. Manipulation of the aortic arch, possible variances in the perfusion times and intraoperative temperature-management, which potentially influence the neurological outcome were not investigated systematically. Furthermore, preoperative hemoglobin levels and anemia rates were not systematically assessed in this study.

## Conclusions

Female sex was identified as an independent predictor for longer ICU length of stay after minimally invasive ascending aortic surgery. Women were found to have a higher risk of perioperative stroke and required more red blood cell transfusion compared to men, despite comparatively shorter operation times. These findings suggest that sex impacts outcome after minimally invasive aortic surgery. Especially for the factors, for which minimally invasive approaches have been shown to be beneficial in the past, i.e. length of ICU stay and transfusion requirements, worse outcomes were observed for female patients undergoing minimally invasive surgery of the ascending aorta compared to male patients. Currently available literature on this phenomenon is by far insufficient: No reports existed to date that investigated the potential impact of sex on minimally invasive ascending aortic surgery. Furthermore, investigations on sex-specific outcomes after cardiac surgery are mainly performed as registry- and database- analyses of conventional, full sternotomy procedures and prone to selection bias. Thus, results cannot simply be extrapolated towards minimally invasive procedures. Nonetheless, as shown in this study, patient sex plays a decisive role for postoperative outcomes after minimally invasive cardiac surgery as well. This should be considered a strong call for more specific research investigating the factors that mediate outcome differences between female and male patients in cardiac surgery.

## Data Availability

Further data used for this research is available from the corresponding author upon reasonable request.
